# Rapid detection of *Mycoplasma synoviae* by loop-mediated isothermal amplification

**DOI:** 10.1007/s00203-014-1063-2

**Published:** 2014-11-21

**Authors:** Olimpia Kursa, Grzegorz Woźniakowski, Grzegorz Tomczyk, Anna Sawicka, Zenon Minta

**Affiliations:** 1 Department of Poultry Diseases, National Veterinary Research Institute, Partyzantów 57 Avenue, 24-100 Puławy, Poland; 2 Department of Poultry Viral Diseases, National Veterinary Research Institute, Partyzantów 57 Avenue, 24-100 Puławy, Poland

**Keywords:** *Mycoplasma synoviae*, Loop-mediated isothermal amplification, Rapid detection

## Abstract

*Mycoplasma synoviae* (MS) remains a serious concern in production of poultry and affects world production of chickens and turkeys. Loop-mediated isothermal amplification (LAMP) of DNA has been recently used for the identification of different economically important avian pathogens. The aim of this study was to develop LAMP for simple and inexpensive detection of MS strains in poultry using specifically designed primers targeting hemagglutin A (*vlh*) gene. The assay was conducted in a water bath for 1 h at 63 °C. The results were visualized after addition of SYBR Green^®^ fluorescent dye. LAMP was specific exclusively for MS without cross-reactivity with other *Mycoplasma* species. The sensitivity of LAMP was determined as 10^−1^ CFU/ml and was 1,000 times higher than MS-specific polymerase chain reaction. LAMP assay was conducted on 18 MS field strains to ensure its reliability and usefulness. This is the first report on LAMP development and application for the rapid detection of MS isolated from chickens. This simple method may be applied by diagnostic laboratories without access to expensive equipment.

## Introduction

*Mycoplasma synoviae* (MS) infections are common and main cause of chronic subclinical upper respiratory disease, infectious synovitis, and airsacculitis in chickens and turkeys (Kleven 2003; Ferguson-Noel and Noormohammadi 2013). Similar to *Mycoplasma gallisepticum* (MG) MS infections have a great economical impact on the commercial poultry industry causing growth retardation, weight loss, and decreased egg production (King et al. 1973; Kleven 2003; Feberwee et al. 2008; Catania et al. 2010; Ferguson-Noel and Noormohammadi 2013). Its exact effect in layer chicken industry is still a current topic of investigations because of a recently found new clinical form called eggshell apex abnormalities described in several countries (Feberwee et al. 2009; Catania et al. 2010; Landman 2014). An effective treatment of its clinical form was successfully treated using tylosin, administered in the drinking water for 5 days (Catania et al. 2010). However, the most preferred method of MS control in poultry flocks is its eradication (Olson et al. 1956; Stipkovits and Kempf 1996; Fiorentin et al. 2003).

Lateral transmission of MS occurs by direct contact and spreads between the birds kept in the same poultry house (Kleven et al. 1975; Ewing et al. 1998; Ferguson-Noel and Noormohammadi 2013). Infection is transmitted via the respiratory tract and usually 100 % of birds become infected. In spite of the clinical form of the disease visible as joint lesions is not always observed, the infected birds remain carriers of MS (Ewing et al. 1998; Kleven 2003). MS infections most frequently occur as a chronic subclinical upper respiratory infection (Landman and Feberwee 2004). Meanwhile, not all chicken flocks showing high antibody levels against MS suffer from infectious synovitis (Jordan 1975; Kleven et al. 1975; Ferguson-Noel and Noormohammadi 2013). Some factors including age of birds, flock size and farming conditions may affect severity of MS in poultry flocks. Taking into account these aspects, rapid and efficient diagnostics of MS is an important issue not only for prevention of clinical disease progress but also infection transmission (Landman 2014).

So far, MS diagnosis has been carried out by bacteriological isolation, serological assays, or polymerase chain reaction and its modification (PCR, multiplex PCR or real-time PCR) (Lauerman et al. 1993; Hong et al. 2004; Hess et al. 2007; Feberwee et al. 2008; Hammond et al. 2009; Raviv and Kleven 2009; Dijkman et al. 2013, Fraga et al. 2013; Reck et al. 2013). The sensitivity of direct MS culturing from affected joints is low and often yields negative results, especially in the sub-chronic stage of the disease (Salisch et al. 1998). Furthermore, direct MS culturing is generally expensive and time consuming because it takes up to 28 days. In serological methods, sera collected from infected birds are commonly analysed for the presence of antibodies by serum plate agglutination (SPA) test, seldom by hemagglutination-inhibition (HI) test or enzyme-linked immunosorbent assay (ELISA) (Ewing et al. 1998; Feberwee et al. 2008).

Although there are many PCR-based methods for detection of MS, they are still laborious, time consuming and require advanced laboratory equipment (Hong et al. 2004; Hammond et al. 2009; Raviv and Kleven 2009; Wetzel et al. 2010; Fraga et al. 2013; Reck et al. 2013).

An advance in detection of many different human and animal pathogens was loop-mediated isothermal amplification (LAMP) developed by Notomi et al. (2000). LAMP depends on auto-cycling strand displacement DNA synthesis performed by *Bst* or *Bsm* DNA polymerase using two or three sets of primers complementary to six or eight different regions in the gene of interest (Fu et al. 2011). LAMP uses a set of inner primers (forward FIP and backward BIP primer), outer primer (including F3 and B3) and forward and backward loop primers (FL and BL) which enable faster formation of loop and hairpin-like structures thus faster detection of specific DNA. Amplification is performed under isothermal conditions of 60–65 °C, while the final products form cauliflower-like structures with multiple overhangs (Notomi et al. 2000; Woźniakowski et al. 2011).

LAMP method has been widely applied for the diagnosis of bacteria like *Shigella spp.*, *Salmonella spp*, *E. coli* (Fu et al. 2011) as well as viral and fungal disease in human and animals (Saito et al. 2005; Huang et al. 2010; Yang et al. 2010; Woźniakowski et al. 2012; Mair et al. 2013; Kakuya et al. 2014). Some of them are economically important poultry pathogens thus LAMP facilitated an improvement in their efficient detection.

The objective of this study was to develop LAMP for the detection of MS without the need of thermal cycler application using water bath or heating block. This is the first report on the development of LAMP for the specific MS identification.

## Materials and methods

### Strains

Seven reference *Mycoplasma spp.* strains purchased from ATCC collection comprising of *M. synoviae* (ATCC 25204), *M. gallisepticum* (ATCC 19610), *M. meleagridis* (ATCC 25284), *M. iowae* (ATCC 33552)*, M. anatis* (ATCC 25524), *M. anseris* (ATCC 49234) and *M. cloacale* (ATCC 35276) were used. Additionally, 18 field isolates of MS collected during the years 2010–2014 were included. These strains were isolated and identified at the Department of Poultry Diseases at the National Veterinary Research Institute (NVRI, Pulawy, Poland).

### DNA extraction

Extraction of genomic DNA from MS reference strains and field isolates was conducted using the QIAamp Mini Kit (Qiagen, Hilden, Germany) following the manufacturer’s instructions. The DNA samples were stored at −70 °C until further analysis.

### LAMP

Based on the *vlhA* sequence of SB5654 MS strain (Accession number: KC506824), six complementary primers were designed using Primer Explorer V4 software (NetLaboratory, Tokyo, Japan) and manual assignment. The primer sequences are listed in Table 1. LAMP reactions were conducted in 15 µl of reaction mixtures which contained: 7.5 µl of Isothermal Mastermix (OptiGene, Horsham, West Sussex, United Kingdom), 10 pM of each inner primer FIP and BIP, 2.5 pM of each outer primer F3 and B3, 5 pM of “loop” primers: LoopF and LoopB primer, 1.5 µl of deionised water and 2 µl of DNA template.

**Table 1:** Sequences of LAMP primers used in the study

Primer	Sequence (5′–3′)	Length (nt)
F3	GGTGATCAAACTCCAGCA	18
B3	TAACCGATCCGCTTAATGC	19
FIP (F1c + F2)	CTCCTGGGTTTCCTGGGTTTCCCTGCTCCAACACCTGG	38
BIP (B1c + B2)	TTGACCCTGTAGAGGCTGCTAGCATCTGCTGTTGTAGTTGT	41
LoopF	TGGGTTTCCTGGATTTGGG	19
LoopB	GCTATTGATGCTGCAACAGAAT	22

F3, forward outer primer; B3, forward backward primer; FIP, forward inner primer (F1C + F2); BIP, backward inner primer (B1c + B2); LoopF, forward loop primer; LoopB, backward loop primer

The reaction mixtures were incubated at the temperature range from 56 to 70 °C for a different time (from 30 min to 105 min) and subsequently heated up to 80 °C for 2 min to inactivate the enzyme. After this step, 1 µl of 1:10 dilution of 10,000× concentrated SYBR Green^®^ (Invitrogen, Paisley, UK) was added to each sample. Results of LAMP were observed by naked-eye, under UV light or after gel electrophoresis in 2 % agarose gels stained with ethidium bromide (0.5 µg/ml). The length of LAMP products was compared to 100-bp DNA Ladder Plus GeneRuler™ (Thermo-scientific, Waltham, Massachusetts, USA). The colour of reaction mixtures in negative controls was orange, while the positives showed green colour or greenish fluorescence. The sensitivity of LAMP was tested using nine 10-fold dilutions (10^6^–10^−2^ CFU/ml) of DNA extracted from MS reference strain (ATCC 25204) with the entire titre 1 × 10^6 ^CFU/ml. The analytical specificity was tested using DNA extracted from six different *Mycoplasma spp*. reference strains.

### PCR

PCR was conducted using MSLF and MssR primers as previously described (Wetzel et al. 2010). Reaction was carried out in a final volume of 25 µl containing 12.5 µl of Taq PCR Master Mix (EurX, Gdansk, Poland), 10 pM of each MsLF and MSsR primer and 7.5 µl deionised water. The thermal profile was 94 °C for 2 min, followed by 35 cycles of 94 °C for 30 s; 55 °C for 30 s; 72 °C for 60 s and final extension at 72 °C for 7 min. The PCR products were separated in 2 % agarose gel stained with ethidium bromide (0.5 µg/ml). The length of PCR products was compared to 100-bp DNA Ladder Plus GeneRuler™ (Thermo-scientific, Waltham, Massachusetts, USA).

## Results

### LAMP conditions

In order to determine the optimal conditions of LAMP, MS reference strain (ATCC 25204) was used as the target template. LAMP assays were incubated under isothermal condition between 56 and 70 °C. The specific colour change, greenish fluorescence and amplicons were observed at 66.3 °C, but in case of field MS strains, the optimal temperature was 63.0 °C. The optimal LAMP duration time was 60 min. Longer incubation up to 105 min had no influence on the final results. Therefore, the final LAMP conditions for all strains was 63 °C for 60 min then 80 °C for 2 min to deactivate the polymerase.

### Analytical sensitivity and specificity of LAMP

**Fig. 1:**
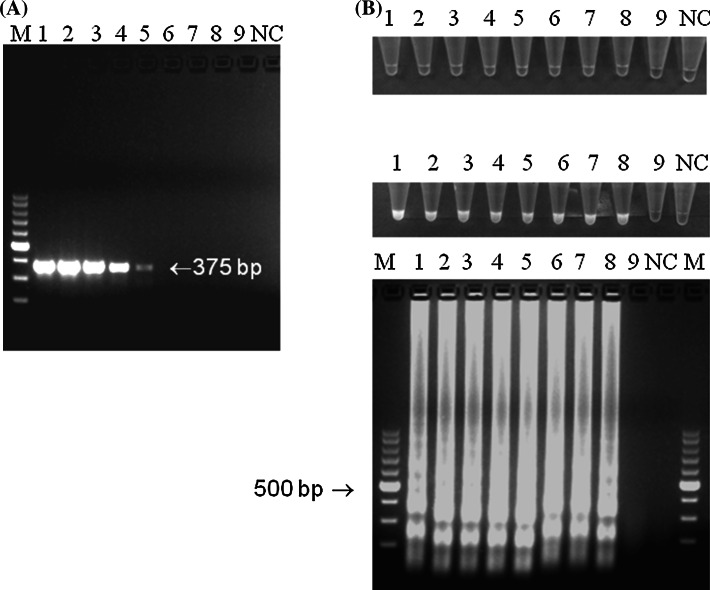
Analytical sensitivity of LAMP and PCR for MS detection. **a** PCR detection of *vlhA* fragment. The product length is 375 bp and was indicated with an *arrow*. **b** LAMP. Visible colour change in MS-positive samples, greenish fluorescence under UV light and specific ladder-like patter after gel electrophoresis. *Descriptions* *M* molecular length marker GeneRuler™ 100 bp DNA Ladder Plus (Thermo-scientific, Waltham, Massachusetts, USA), 10-fold dilutions of reference *M. synoviae* strain (ATCC 25204): *1* 10^6^ CFU/ml, *2* 10^5^ CFU/ml, *3* 10^4^ CFU/ml, *4* 10^3^ CFU/ml, *5* 10^2^ CFU/ml, *6* 10^1^ CFU/ml, *7* 10^0^ CFU/ml, *8* 10^−1^ CFU/ml, *9* 10^−2^ CFU/ml, and *NC* negative control—DNA extracted from non-inoculated growth medium

**Fig. 2:**
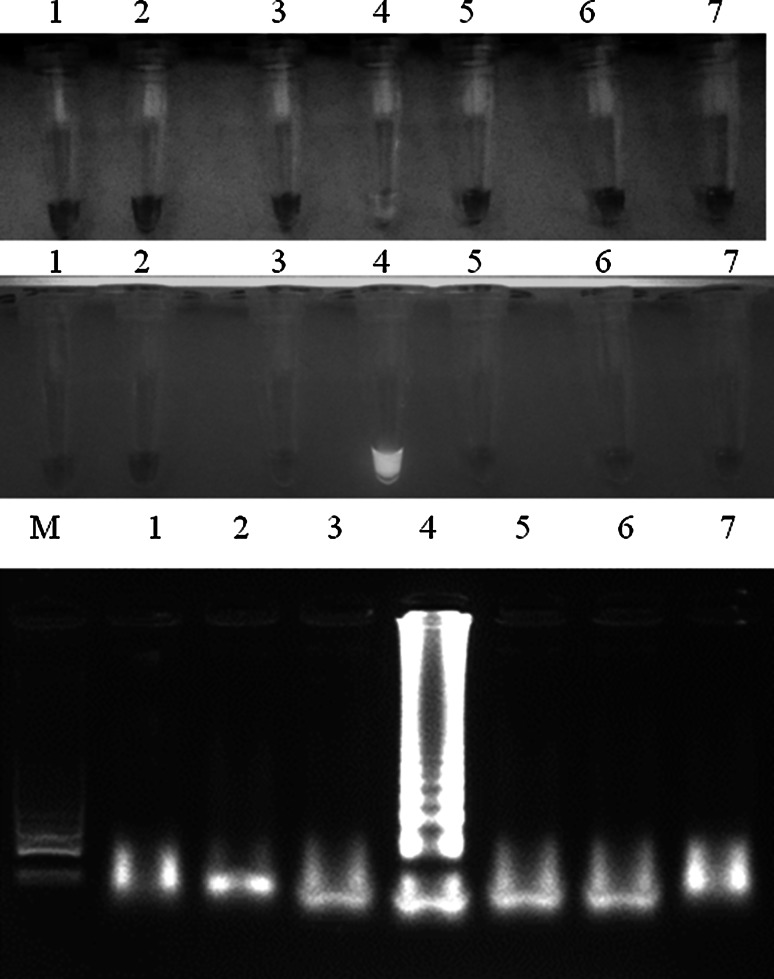
Analytical specificity of LAMP for MS detection. Visible colour change in MS-positive samples, greenish fluorescence under UV light and specific ladder-like patter after gel electrophoresis. *Descriptions* *M* molecular length marker GeneRuler™ 100 bp DNA Ladder Plus (Thermo-scientific, Waltham, Massachusetts, USA), *1* *M. gallisepticum* (ATCC 19610), *2* *M. meleagridis* (ATCC 25284), *3* *M. iowae* (ATCC 33552)*, 4* *M. synoviae* strain (ATCC 25204), *5* *M. anatis* (ATCC 25524), *6* *M. anseris* (ATCC 49234) and *7* *M. cloacale* (ATCC 35276)

The analytical sensitivity of LAMP showed that the detection limit was 10^−1^ CFU/ml of MS reference strain (ATCC 25204) and was 1,000 times higher in comparison with conducted PCR (Fig. 1). LAMP was specific only for the reference strain of MS. No colour change, fluorescence nor ladder-like pattern was observed in LAMP samples containing other species of *Mycoplasma* (Fig. 2). This indicated high analytical specificity of established LAMP assay for the selective detection of MS.

### Field samples of MS

**Fig. 3:**
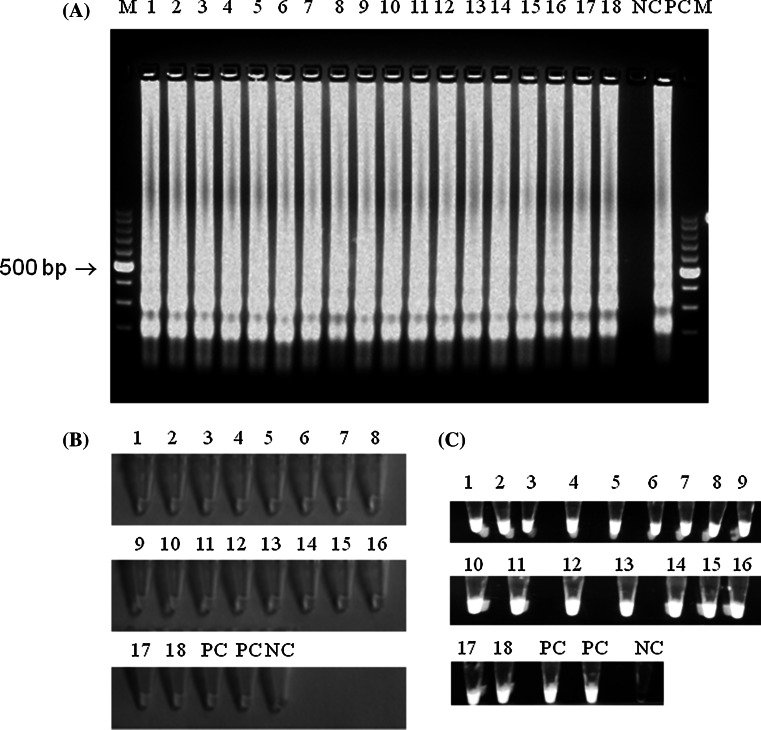
Analysis of field MS isolates by LAMP. **a** Ladder-like pattern in MS-positive samples after gel electrophoresis, **b** visible colour change in MS-positive samples, **c** greenish fluorescence under UV light. *Descriptions* *M* molecular length marker GeneRuler™ 100 bp DNA Ladder Plus (Thermo-scientific, Waltham, Massachusetts, USA), 1–18 field MS isolates, *NC* negative control—DNA extracted from non-inoculated growth medium, *PC* positive control DNA of reference *M. synoviae* strain (ATCC 25204)

All 18 field isolates of MS were positive in conducted MS-specific PCR (data not shown). The similar results were obtained using developed LAMP. The colour change of reaction mixtures and specific ladder-like pattern was detected in reference MS strain as well as in all 18 field isolates (Fig. 3). This indicated on the capability of LAMP to detect both reference and field MS isolates.

## Discussion

Until now, MS culturing methods, serological assays and conventional PCRs or real-time PCRs are useful in detection of MS strains (Lauerman et al. 1993; Hong et al. 2004; Hess et al. 2007; Feberwee et al. 2008; Dijkman et al. 2013). However, culturing is very laborious, time consuming and requires specific growth medium and incubation (Ratliff et al. 2014). PCR is highly specific and sensitive technique but requires at least few hours, and electrophoresis is needed to visualize the results. Indeed, it has been previously presented that multiplex PCR is a valuable tool for the detection or differentiation of MG and MS in chickens (Garcia et al. 1995; Hess et al. 2007; Hammond et al. 2009; Wetzel et al. 2010; Fraga et al. 2013). Moreover, using PCR and PCR–RFLP method targeted on single-copy domain of the N-terminal *vlhA* region, it is possible to differentiate field MS isolates from MS-H vaccine strain (Bayatzadeh et al. 2014). Based on 16S-23S rRNA intergenic spacer region, it was also possible to quantify MS copy number in samples collected from chicken joints using specific qPCR technique (Raviv and Kleven 2009; Dijkman et al. 2013). An attractive alternative to traditional methods of MS detection is LAMP which is simple to set and requires no additional thermal cyclers (Woźniakowski et al. 2012). Previously, LAMP has been applied for detection of *M. hyorhinis in* specimens of bronchoalveolar lavage fluid (BALF) from pigs (Du et al. 2013). The analytical sensitivity of LAMP reached ten DNA copies per ml that was 1,000 times higher than in case of conventional PCR. Indeed, in our study, LAMP showed to be very sensitive detecting 10^−1^ CFU/ml of standard MS strain. Other study published by Mair et al. (2013) showed LAMP usefulness for detection of *M. mycoides* subsp. *mycoides* as the technique that may be run on a battery-driven mobile device. The efficacy of LAMP was also confirmed in the study on detection of *M. pneumoniae* (MP) as the lower respiratory tract pathogen in children (Ratliff et al. 2014). Similar example of LAMP application for the detection of *M. wenyonii* in cattle was presented by Song et al. (2012). The conducted study on development of LAMP for MS identification provides an additional evidence of the usefulness of this technique as a rapid and very sensitive method for *Mycoplasma ssp*. detection.

## Conclusions

In conclusion, the developed LAMP showed to be a powerful and robust method for the specific and selective detection of *M. synoviae*. Using specific region of *vlhA* gene, it was possible to selectively amplify DNA of MS without cross-reactions in other samples containing DNA of *Mycoplasma spp.* This method presents an useful alternative for local veterinarians and small veterinary laboratories without an access to PCR technique.

## Abbreviations

AIV: Avian influenza virus
ATCC: American Type Culture Collection
B3: Forward backward primer
BIP: Backward inner primer (B1c + B2)
*Bst*: Thermostabile enzyme isolated from *Bacillus smithii* bacteria
CAV: Chicken anaemia virus
CFU: Colony-forming unit
F3: Forward outer primer
FIP: Forward inner primer (F1C + F2)
HI: Hemagglutination-inhibition test
IBV: Infectious bronchitis virus
LoopF: Forward loop primer
LoopB: Backward loop primer
NDV: Newcastle disease virus
MS: *Mycoplasma synoviae*
MG: *Mycoplasma gallisepticum*
PCR: Polymerase chain reaction
SPA: Serum plate agglutination
*vlh*: Hemagglutin A gene of MS
